# Spatial Relations between Coccoliths and Their Confining Membrane During Crystal Morphogenesis

**DOI:** 10.1021/jacs.6c02151

**Published:** 2026-03-24

**Authors:** Emanuel M. Avrahami, Dmitry Karpov, Lior Aram, Nadav Elad, Razi Safadi, Irit Rosenhek-Goldian, Xiao-Meng Sui, Diede de Haan, Neta Varsano, Sidney R. Cohen, Igor Zlotnikov, Assaf Gal

**Affiliations:** † Department of Plant and Environmental Sciences, 34976 Weizmann Institute of Science, Rehovot 7610001, Israel; ‡ 27015 Université Grenoble Alpes, CEA, IRIG, MEM, NRX, Grenoble 38054, France; § European Synchrotron Radiation Facility, Grenoble 38043, France; ∥ Department of Chemical Research Support, Weizmann Institute of Science, Rehovot 7610001, Israel; ⊥ B CUBE - Center for Molecular Bioengineering, 9169 Dresden University of Technology, Dresden 01307, Germany

## Abstract

Coccoliths are multicrystalline calcite structures formed by microalgae within an intracellular vesicle. The morphology of each crystal is complex, and recent studies postulate that coccolith morphogenesis is regulated by the bounding membrane of its vesicle. However, the limited information about the native-state organization within the cell makes it difficult to understand which structural aspects of the membrane are responsible for morphogenesis. Here, we examined the vesicular environment during the formation of *Calcidiscus leptoporus* coccoliths, using advanced cryo-electron microscopy and X-ray fluorescence tomography. Our findings show two distinct types of crystal surfaces that persist during coccolith development: flat and curved, which differ also in roughness. Interestingly, even though both types of surfaces have variable degrees of membrane confinement, they are separated by distinct “bands” of intimate contact between the crystals and the membrane. We propose that these “bands” serve as boundaries, creating subcompartments within the coccolith vesicle that regulate crystal growth and its cessation. These results suggest that the coccolith vesicle membrane maintains distinct and functional chemical environments on the nanometer scale.

## Graphical abstract

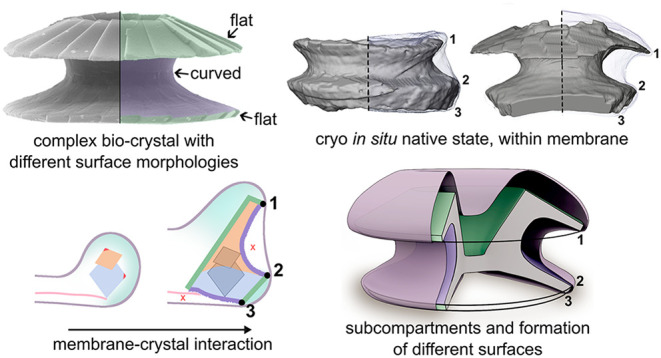

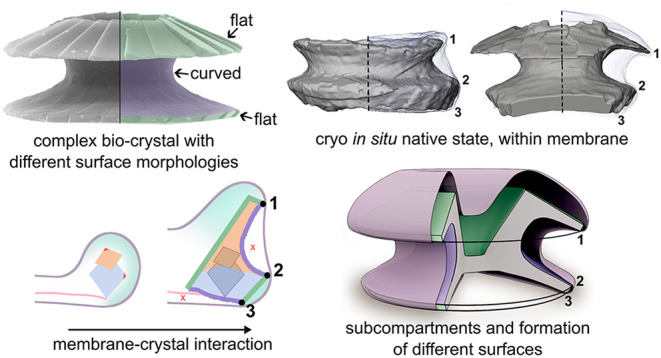

## Introduction

Coccoliths are microscopic multicrystal assemblies made of calcite (CaCO_3_). They are produced at ambient conditions by a widespread group of marine unicellular algae called coccolithophores.^1,2^ Every coccolithophore species fabricates its own coccolith morphology, ranging from simple polyhedral shapes to complex interlocking hierarchical structures with spines, bridges, and spiral patterns. Each coccolith is produced intracellularly, and upon maturation is exocytosed to the cell surface where several-to-dozens of coccoliths cover the cell to form an external shell (Figure 1A).^3^ Many mature coccolith crystals appear to have two types of external surfaces, being either flat, so-called “crystallographic,” or curved and undulating with no clear crystallographic characteristics. Consequently, coccoliths represent among the most intricate and morphologically diverse biogenic crystalline structures, embodying control over crystal growth that surpasses current synthetic fabrication capabilities. It is still openly debated whether their morphological diversity is the outcome of crystallographic growth with modified kinetics or of other processes.^4–10^

**Figure 1:**
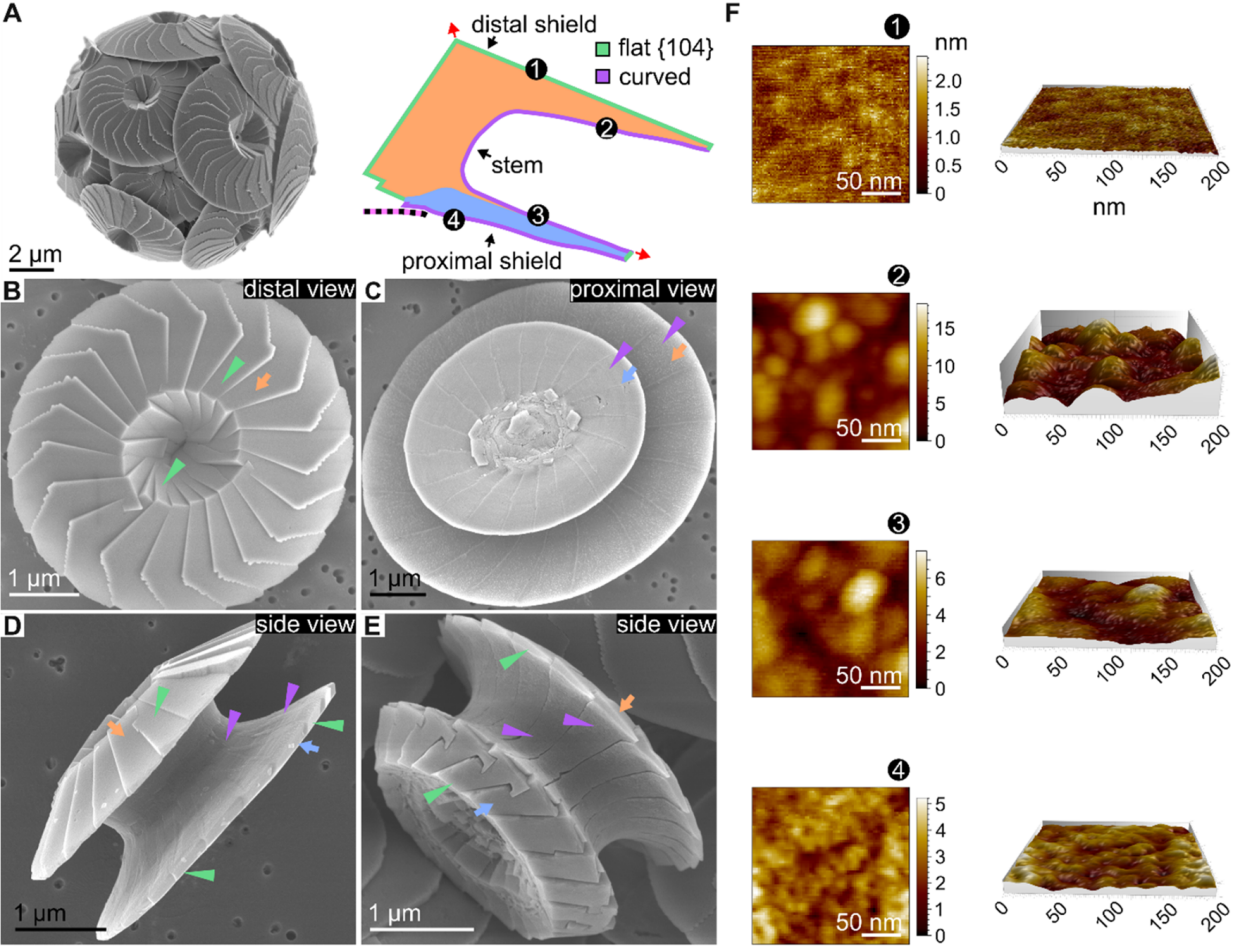
Different surface topographies of *Calcidiscus leptoporus* coccolith crystals. (A) Left: SEM image of a coccolith-covered cell. Right, schematic cross-section of half of a mature coccolith. The distal shield and stem, made of V-units, are colored orange, and the proximal shield, made of R-units, is colored blue. Red arrows indicate the *c*-axis direction of each unit. Two distinct external surface-types are highlighted: flat {104} (green outline) and curved (purple outline). The base plate is depicted in pink and black. Numbered annotations indicate the four different surfaces analyzed with AFM scans in panel (F). (B–E) SEM images of different regions in coccoliths. Flat and curved surfaces are indicated with green and purple arrowheads, respectively. V-units and R-units are indicated with orange and blue arrows, respectively. (F) Representative AFM scans of the different surfaces indicated in panel (A). Each scan is 200 × 200 nm^2^ in area. Left, 2D plan view of a scan with color scale showing height; right, 3D projections of the respective 2D scans on the left. All 3D projections have their *z*-axis set to the same scale. All values are in nanometers. See more details in Figures S1 and S2.

The formation of coccoliths takes place within a specialized membrane-bound, Golgi-derived, compartment known as the coccolith vesicle (CV).^11^ Prior to crystallization, a thin organic disc called the base plate (BP) forms inside the CV.^12^ Then, crystal nucleation occurs along the periphery of the BP, resulting in alternating rhombohedral units known as V and R; these units are named for the orientation of their crystallographic *c*-axis relative to the BP surface—vertical or radial, respectively.^13^ Following nucleation, the crystal units undergo growth and morphogenesis to form their highly anisotropic structures. During coccolith development, the CV membrane is dynamic, undergoing morphological changes (i.e., it does not persist as a simple ellipsoid vesicle) as well as expanding in surface area to accommodate the growing coccolith within.

The mechanisms by which coccolithophores regulate crystal morphology at high precision remain unresolved. Recent studies showed that coccolith crystals are shaped through anisotropic growth of only the thermodynamically stable, {104}-rhombohedral, facets of calcite.^14–16^ The underlying reason for this growth has been proposed to be the presence of different locations in the CV with distinct chemical environments.^14^ The crystals grow wherever the environment is conducive to growth; however, where building blocks are lacking or conditions do not promote growth, curved surfaces emerge due to incomplete formation of facets.^14^

Several recent in situ studies used high-resolution cryo-electron tomography to image both crystals and membranes in their native state.^17–19^ These studies were conducted on the species *Emiliania huxleyi* and *Pleurochrysis carterae* (renamed *Gephyrocapsa huxleyi* and *Chrysotila carterae*), which have thin crystals showing only minute crystallographic surfaces throughout their development. These works suggested two modes by which restriction of building blocks could occur: (i) high membrane proximity to a curved surface, of only tens of nanometers, which acts as a “molding” agent for the crystal; (ii) ion restriction due to limited transport/diffusion, which can leave incomplete faces, similar to dendritic growth seen in ice crystals.^20^

In this study, we investigated the intracellular development of coccoliths of *C. leptoporus* using a suite of advanced microscopy techniques. This species has large coccoliths with pronounced flat and curved surfaces, whose morphological characteristics were previously characterized.^14^ Our examination provided spatial information about the interactions between the CV membrane and the crystals. The results revealed that while both flat and curved surfaces exhibit variable degrees of membrane confinement, they are separated by specific sealing “bands” where crystals and membrane meet closely, creating subcompartments within the coccolith vesicle that control growth patterns. These results challenge the view of the membrane as a molding surface while providing an alternative perspective on crystal morphogenesis through compartmentalized conditions for growth control.

## Results

To analyze the properties of the different coccolith surfaces, we isolated and imaged coccoliths from the species *C. leptoporus* (Figure 1). Each multicrystal coccolith could be schematically described as two parallel disks (shields) connected by a tube (stem). In detail, these elements are (i) the distal shield, composed of V-units; (ii) the proximal shield, composed of R-units; (iii) the stem, which connects the two shields, and is also part of the V-units. The terms “distal” and “proximal” refer to the orientation of an exocytosed coccolith relative to the cell body (Figure 1A). Scanning electron microscopy (SEM) images show two surface-types: one, having a flat-and-smooth topography that characterizes the distal side of the distal shield and the growing faces of the proximal shield, and the second, having curved-and-rough topography, seen in all other surfaces (Figures 1B–E and S1).

For a quantitative characterization of the local surface roughness of each coccolith shield-surface, we used an atomic force microscope (AFM) situated inside an environmental SEM (ESEM) purged with N_2_ gas to reduce charging, (Figures 1F and S2). To prepare the samples, isolated and cleaned coccoliths were blotted on a polycarbonate membrane filter, which was placed on an aluminum stub using a conductive adhesive. The correlative imaging in ESEM allowed us to locate coccoliths and choose specific surfaces. The AFM measurements show that the distal side of the distal shield (surface 1 in Figure 1A,F) exhibited the smoothest surface, with peak-to-peak (p–p) height variations under 3 nm (mean Root Mean Square, or RMS, roughness 0.45 ± 0.1 nm over 200 × 200 nm^2^ regions), while its proximal side (surface 2 in Figure 1A,F) displayed the highest roughness, showing variations of approximately 12 nm p–p (mean RMS roughness 2.8 ± 0.5 nm). The proximal shield demonstrated intermediate roughness on both sides (surfaces 3 and 4 in Figure 1A, F), with height variations ranging up to 8 nm p-p (mean RMS roughness values of 1.5 ± 0.4 and 1.1 ± 0.3 nm for the proximal and distal side, respectively). Altogether, the SEM and AFM analyses revealed distinct variations in surface topography, showing that the crystallographic surfaces are smoother than the curved surfaces. This data supports the hypothesis that different regions of the coccolith structure form under distinct growth regimes.

To examine the formation environment of intracellular coccoliths in situ, we used a suite of cryo-electron microscopy techniques. First, actively calcifying cells were high-pressure-frozen and freeze-fractured for cryo-SEM imaging. The images show the overall cellular organization, including intracellular coccoliths within the CV membrane (Figure 2A,B). Interestingly, along the stem region—an area previously suggested to be the outcome of molding—the membrane is located further from the crystals compared to *E. huxleyi* and *P. carterae*,^17,18^ at over 100 nm (Figure 2B, yellow arrow). In contrast, at growing crystal fronts, the membrane seems to be almost touching the crystals (Figure 2B, black arrows).

**Figure 2:**
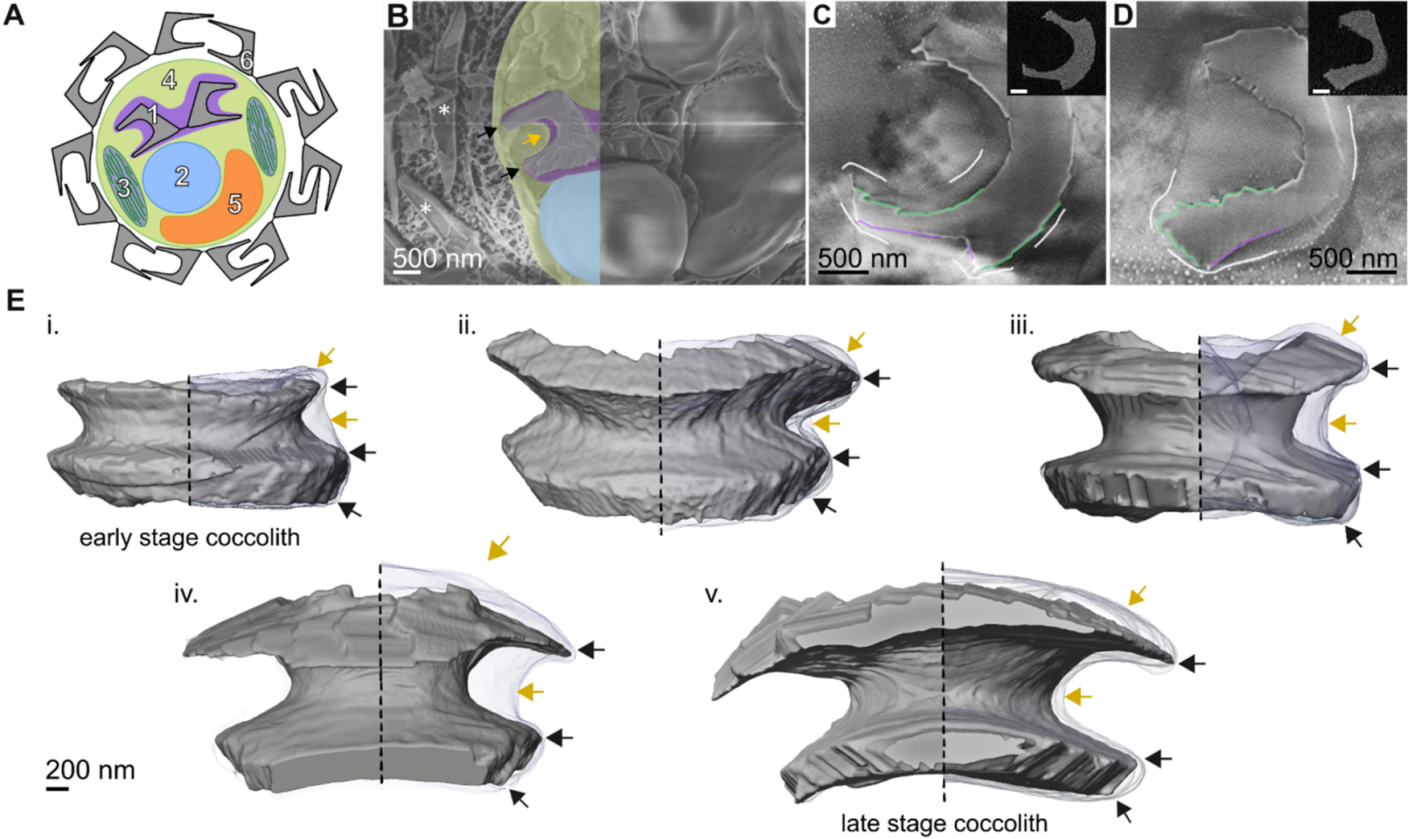
Variable proximity of the CV membrane to the growing crystals. (A) Schematic cross-section of cellular components (not to scale): (1) intracellular coccolith inside a CV; (2) nucleus; (3) chloroplasts; (4) cytosol; (5) vacuole; (6) extracellular coccolith. (B) cryo-SEM image of a cell harboring an internal coccolith (similar to stage v in panel (E)). For ease of view, a part of the cell body is artificially colored in colors matching those in panel (A). (C and D) Two slices from the same cryo-FIB-SEM data set (stage ii in panel (E)). Some elements are highlighted to guide the eye: white, CV membrane; green, flat crystal surfaces; purple, curved surfaces. Insets show backscattered electron images with a strong signal from the coccolith crystals. Inset scale bars, 500 nm. (E) 3D volume rendering of five coccoliths from different growth stages acquired using cryo-FIB-SEM; stages are ordered top left to bottom right (indicated by roman numerals) and are shown to scale. For each coccolith, the membrane engulfing the crystals is shown in the right half. In panels (B) and (E), points of regular high proximity between the membrane and the crystals are indicated by black arrows, whereas areas of variable proximity are indicated by yellow arrows.

To get a 3D visualization of the CV membrane relative to the crystals during different stages of coccolith growth, we implemented in vivo cryo-focused ion beam milling with SEM imaging (cryo-FIB-SEM) on vitrified cells in the near-native state. This allows for a high degree of three-dimensional structural preservation of cellular components in their hydrated environment without the need for fixatives or heavy metals and enables imaging data sets of large samples (e.g., entire cells) which can then be aligned to produce a 3D volume. For this, actively calcifying cells were stripped of their external coccoliths and immediately vitrified in liquid ethane. Using the FIB, we first performed rough milling through the cells until we encountered a coccolith within a CV. Identification of the CV was enabled by the use of the energy selective backscattered electron (ESB) detector, which revealed the location of intracellular coccoliths due to the high z-contrast of the Ca ions in the crystals (see insets in Figure 2C,D). Upon reaching an internal coccolith, we switched to a finer milling regime to perform slice-and-view and collected images using the in-lens electron detector. Image quality was sufficient to detect both the coccolith and the CV membrane (Figures 2C,D and S3). We then aligned the images, segmented the coccoliths and the membrane, and visualized them in 3D (Figure 2E).

Examination of the various growth stages shows variability in the distance of the CV membrane from the crystals, ranging from tens of nanometers to hundreds of nanometers. It is evident that the CV membrane envelopes the overall shape of the coccolith, but it does not conform tightly to the evolving crystal morphology. Notable exceptions, however, arise from all data sets, where locations of regular high proximity are seen in the external sides of both shields. Remarkably, these locations form continuous bands along crystal edges where transitions from a flat to curved surface occur (Figure 2E, black arrows; depicted as junctions between green and purple surfaces in Figure 1A). Consequently, there appears to be a division of the CV lumen to different subcompartments. The observation that the CV membrane is not tightly mated with the curved-and-rough surfaces suggests that these morphological features are not the direct outcome of membranal molding via a physical block.

Although the cryo-FIB-SEM data sets contain information on the entire CV, they are unable to capture nanometer-scale details. Therefore, we employed high-resolution cryo-electron tomography (cryo-ET) for enhanced structural information on specific regions in the CV. Using a cryo-FIB, we milled ∼200 nm thick lamellae in vitrified cells and then collected transmission electron microscope (TEM) tomography data from regions of interest, where both crystal surfaces and CV membrane are seen (Figures 3 and S4). The data was then reconstructed and visualized in 3D.

**Figure 3:**
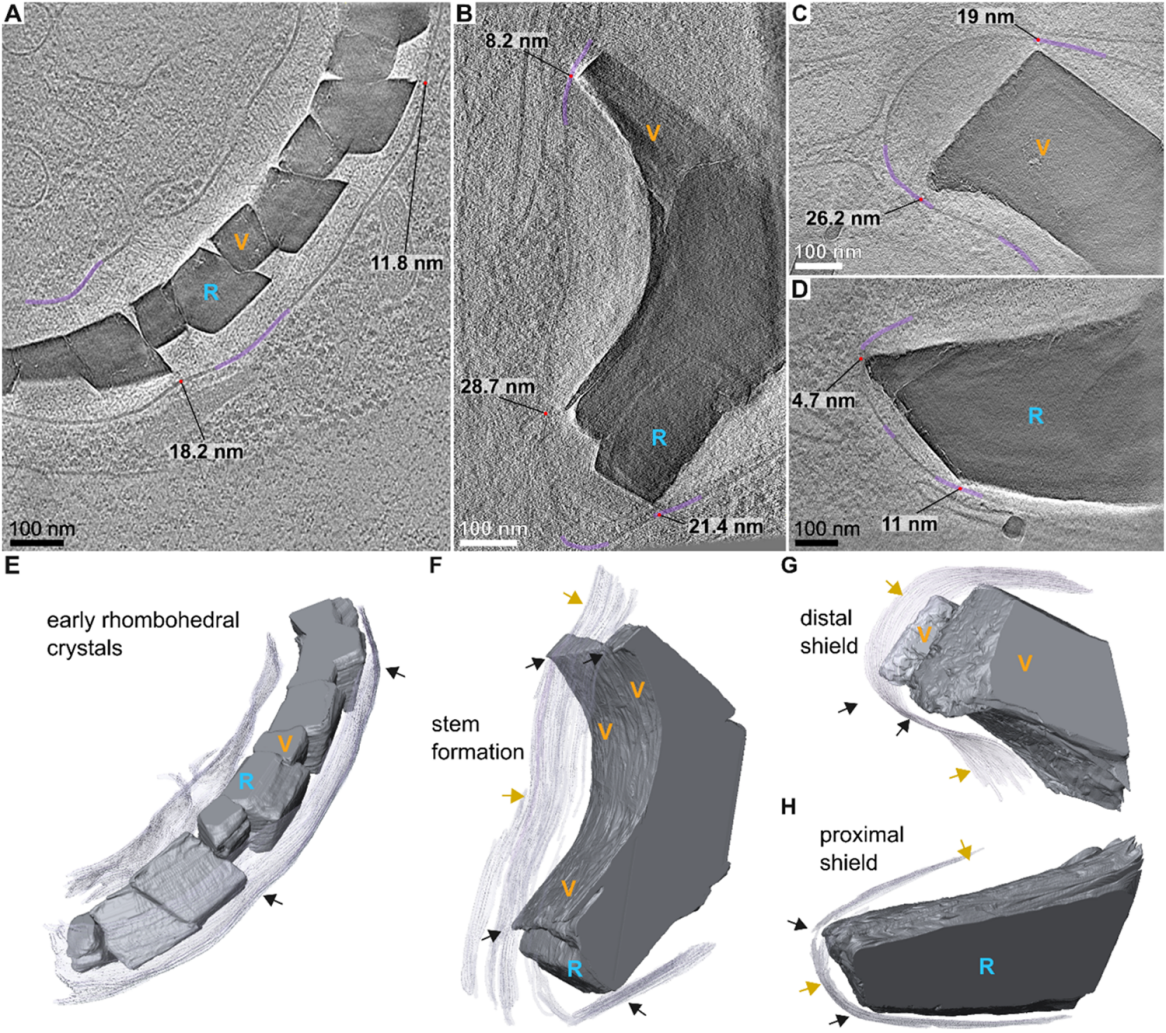
High-resolution cryo-ET shows membrane positioning along crystals. (A–D) Slices from reconstructed TEM tomograms, showing coccolith crystals and CV membranes. Early crystals (A) have crystallographic surfaces, and the CV membrane loosely surrounds them. In intermediate stage (B), the membrane is in the proximity of transition areas where crystallographic surfaces meet the curved surfaces of the stem region. Distances to the membrane in these areas are indicated. In more mature stages, this trend continues: Panel (C) shows a more developed distal shield, where radial growth has begun, and panel (D) shows an extended proximal shield. Throughout (A) to (D), the CV membrane is highlighted in purple, and crystal units are indicated by color (orange, V-units; blue, R-unit). (E–H) Corresponding 3D volume renderings of the tomographic slices in panels (A) to (D), respectively. Points of high proximity between the membrane and the crystals are indicated by black arrows, and areas with variable distances are indicated by yellow arrows.

At an early growth stage in which both V- and R-units are delineated by crystallographic {104} facets (Figure 3A,E), the CV membrane is voluminous, with distances of ∼100 nm from the crystals. We note that points of tighter proximity, down to ∼10 nm can also be seen, yet these do not appear to display regularity. More mature stages, where crystals are anisotropic and the stem region is already developed as well as the shields, mirrored the observations from the cryo-FIB-SEM data (Figure 3B–D,F–H). The distances of the membrane in the variable locations (yellow arrows) range greatly, from ∼20 nm to exceeding 100 nm. Points of regular high proximity (black arrows), ranging from only a few nanometers to ∼30 nm, were detected in the borders between flat-and-smooth and curved-and-rough surfaces. These observations, which are detected in all imaging methods used, suggest that the CV membrane has a tendency to be very close to specific points in the crystals, such that two types of functional “subregions” are formed within the CV. In one region, crystal growth propagates, resulting in flat and smooth surfaces, while in the other region, crystal growth is inhibited and curved surfaces with rough topography prevail. Previous studies have suggested that the cytoskeleton within the cell may be responsible for coccolith morphogenesis,^18,21,22^ yet we detected no cytoskeletal elements (fibers or filaments) in close contact to the CV membrane. This implies an indirect role of the cytoskeleton, which awaits further elucidation.

A careful observation of the high-resolution cryo-ET data showed that the CV membrane is lined with electron-dense particles that face the inner lumen of the CV (Figure 4A–D). The densities can appear as a continuous layer or as distinct globular entities (possibly depending on the quality of the data set), with a diameter of ∼ 5–10 nm, extending ∼10–15 nm from the inner leaflet of the membrane bilayer and separated by a thin gap from it. A previous study in *P. carterae* detected similar looking densities in the luminal side of the CV, showing a structure of a globular “head” connected by a “stalk,” which were suggested to be ATPase complexes based on the overall dimensions.^18^

**Figure 4:**
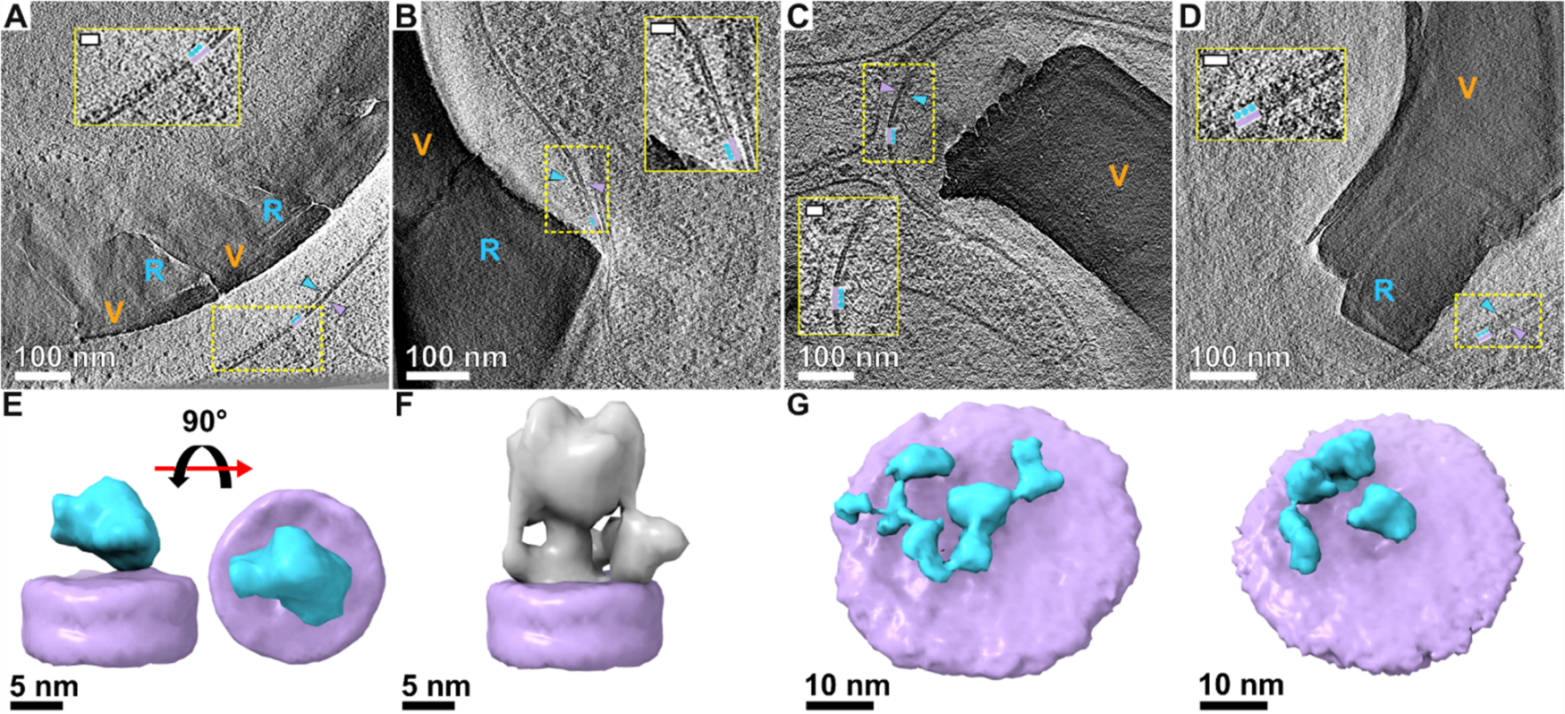
Macromolecular complexes line the CV membrane at different locations. (A–D) Slices from reconstructed tomograms, showing coccolith crystals (unit type is indicated with V or R), CV membrane, and membrane-bound complexes. The complexes (blue arrowheads) along the CV membrane (purple arrowheads) face the luminal side of the vesicle. As a guide to the eye, schematic coloring of the particles and of the membrane is shown, in blue and purple, respectively. Insets are magnifications of the dashed rectangular areas. Inset scale bars: 20 nm. (E) 3D reconstruction (using the same color code) from subtomogram averaging of the membrane-bound complexes. Left, side view; right, top view (seen from inside the CV lumen). This structure was cropped from the central part of the map shown in right panel of (G). (F) ATP synthase structure (PDB 3J9T),^23^ low-pass filtered to 4 nm, is shown for scale and morphological comparison. (G) Two 3D classes rendered with a larger mask compared to the map in panel (E). Neighboring complexes are observed adjacent to the central complex, indicating clustering.

We attempted structural elucidation of these complexes using subtomogram averaging (Figures 4E,G and S5). In this method, similarly looking macromolecular complexes are boxed out of the reconstructed tomograms, aligned, classified, and averaged together. The resulting 3D maps represent the main structural populations with higher contrast and better resolution than the complexes in the raw tomograms. Applying subtomogram averaging to a data set of 716 membrane-bound complexes, resulted in an average density with an overall globular shape, containing an attachment point to the membrane that is offset from the center of the particle (Figure 4E). The resolution of the map was mainly limited by the small data set and possibly by structural heterogeneity. Importantly, comparing the dimensions of the resulting map to a known structure of an ATPase complex (Figure 4F),^23^ shows that it is about half the height of a full ATPase above the membrane, hence challenging the hypothesis that the CV membrane is lined with ATPases, or at least, not the full ATPase complex.^18^ As seen in Figure 4G, the 3D classes included density for several neighboring complexes, apart from the central complex, which was the focus of alignment. This indicates that the complexes are clustered in 2D arrays with some degree of consistency in spacings and relative orientations. It is possible, and yet to be determined, if these proteins are involved in ion delivery at specific regions of the CV or in the physical behavior of the membrane, for example inducing undulation, influencing fluidity or even tethering it to the crystals.^18^

We complemented the various structural data with compositional measurements of the solution inside the different areas of the CV. This is challenging since very high spatial resolution (given the close proximity of the CV membrane to the crystals) and high sensitivity to solutes (given the strong signal emanating from the bulk crystal) are required to be detected inside the cell. For this, we used nano-X-ray fluorescence (nano-XRF) tomography—an advanced synchrotron technique for 3D visualization of chemical mapping that can achieve down to 7 nm spatial resolution in synthetic samples.^24^ When implemented under cryogenic conditions (cryo-nano-XRF), the same general strategy can preserve cells close to their native state and help mitigate radiation damage during acquisition.^25^

We vitrified actively calcifying *C. leptoporus* cells on graphene-coated silicon nitride windows and performed cryo-nano-XRF tomography at the European Synchrotron Radiation Facility (ESRF) ID16A Nanoimaging beamline (Figure 5). We were able to obtain 3D elemental maps of a cell containing an intracellular coccolith as well as other compositionally distinct organelles (Figures 5A,B and S6). Visualization of the Ca signal indicates a thin layer with a low Ca content that surrounds the high Ca signal of the coccolith (Figure 5C). This low Ca signal mirrors the shape of the CV as observed with previous methods (Figure 2); thus, we assign it to soluble Ca in the CV lumen. Importantly, the Ca distribution is not homogeneous around the crystals, with areas of elevated Ca levels extending farther from the crystals at specific locations (Figure 5D–F). These regions, where an elevated Ca signal (above background Ca levels in the cell) was detected more than 100 nm away from the crystals, were observed atop the center of the distal shield and close to the stem region, yet, not near the growing front of the shields. This distribution agrees with the compartmentalization observed by cryo-ET (Figure 3).

**Figure 5:**
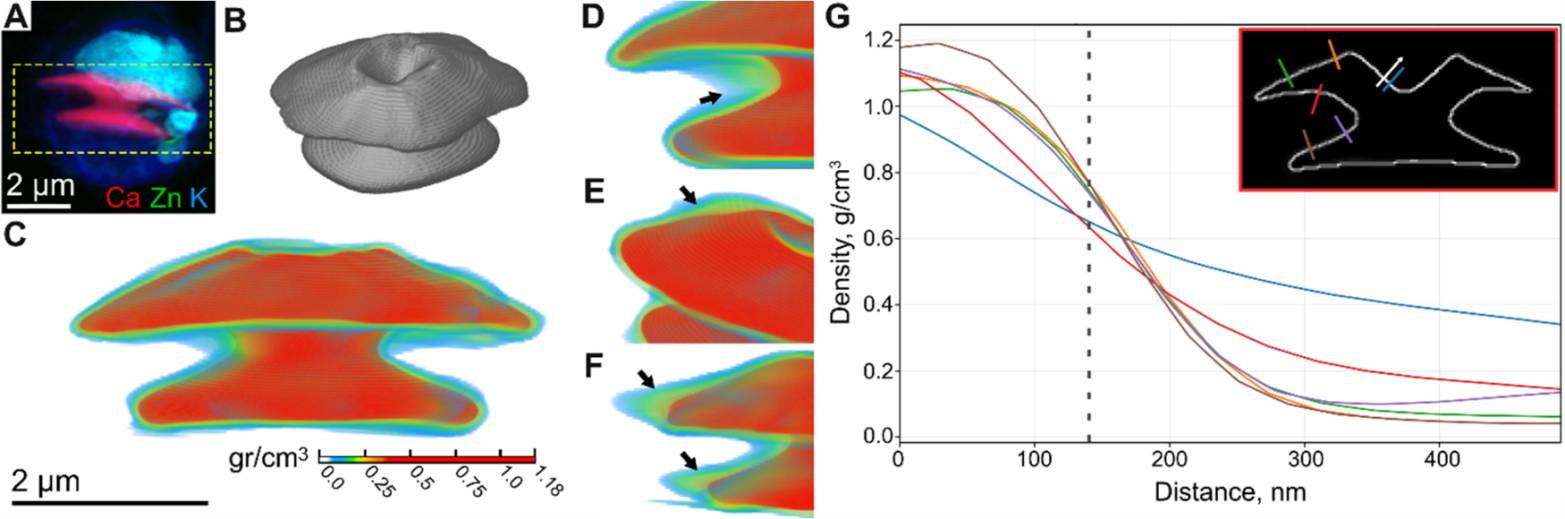
Cryo-nano-XRF tomography of an intracellular coccolith. (A) Composite color image of 2D cryo-nano-XRF maps of Ca, Zn, and K acquired at a 30 nm pixel size. The image shows a cell harboring an intracellular coccolith (Ca signal, red). The outline of the cell and some of its ultrastructure could be visualized using other elements (Zn, green; K, blue). Dashed box indicates region of interest that was used for subsequent cryo-nano-XRF tomography. (B) 3D rendering of the coccolith in panel (A), after tomographic reconstruction of Ca projections acquired at 35 nm pixel size. (C) 3D volume visualization of the Ca signal from the reconstructed XRF tomogram. The values coming from the bulk crystal (red) were visually saturated so that the lower concentrations in the lumen (blue-to-red gradient) could be displayed. (D–F) Magnifications of areas where Ca levels are inhomogeneous and detected away from the crystals (arrows). (G) Ca line profiles measured across the coccolith boundary at multiple positions. Profiles were extracted along outward-pointing surface-normal lines (from inside the CV lumen toward the exterior). Dashed line indicates the position of the edge of the coccolith’s surface, at 140 nm, determined by the infliction point in the profile. Inset shows the locations and orientations of the sampled line segments used to generate the profiles. White arrow is an example of line scan direction.

Analysis of the Ca signal revealed a concentration gradient (0.1–0.5 g/cm^3^) surrounding the coccolith (Figure 5C–F). Although edge effects limit quantification near the crystals, line profile measurements (Figure 5G) show the differential decay of the Ca signal. This suggests that in specific locations, such as the distal side of the distal shield, the signal reflects contributions from soluble Ca rather than the crystals alone. These results demonstrate that soluble Ca can be detected with high sensitivity and spatial resolution, strengthening the scenario of compartmentalized regions within the CV.

## Discussion

In this study, we conducted an in-depth investigation of the spatial relationship between the CV membrane and the crystals in *C. leptoporus* during coccolith development. Through a combination of advanced microscopy techniques including AFM-SEM, cryo-FIB-SEM, high-resolution cryo-ET, and cryo-nano-XRF, we were able to visualize the close-to-native-state interactions between the growing coccolith crystals and their confining vesicle membrane. The use of these complementary techniques allowed us to overcome the limitations of each individual method—the high-contrast but lower resolution of SEM and FIB-SEM, and the high-resolution but limited field of view of cryo-ET and AFM—and to comprehensively characterize the developmental environment of coccoliths from the nanometer to the micrometer scale. Specifically, complete 3D visualization and compositional analysis of the CV and its membrane allowed us to differentiate between variable and constant crystal-membrane interactions.

Our SEM and AFM analyses showed variations in surface topography across different regions of the coccolith. The distal side of the distal shield exhibited a flat and smooth surface belonging to the crystallographic {104} habit. In contrast, its proximal side and both sides of the proximal shield displayed higher roughness, indicative of curved, undulating, noncrystallographic surfaces. In other organisms, similar rough surface features were related to maturation via a transient amorphous phase,^26^ but no evidence for this pathway exists in coccoliths.^18,19^ Our observations support the hypothesis that different surface morphologies reflect distinct physicochemical regimes.^14^ The flat crystallographic surfaces likely develop under conditions favorable for crystal growth, where building blocks are readily available for continuous and ordered deposition along thermodynamically stable planes. Conversely, rough surfaces may emerge in environments where crystal growth is limited or inhibited due to reduced availability of building blocks.^17,20,27^ This distinction reconciles previous works that reported both on atomically flat surfaces in some cases and intricate morphologies of others,^8–10,28–30^ suggesting that local conditions in the subregions of the coccolith vesicle dictate small-scale surface topography and large-scale flatness/undulation.

Cryo-nano-XRF analysis enabled us to examine intravesicular Ca concentrations with high sensitivity and spatial resolution. Although no clear relationship was immediately apparent between composition and growth behavior, we observed luminal Ca within the vesicle, with the highest Ca concentrations occurring at the center of the distal shield’s distal side (Figure 5G, blue curve) and near the stem region (Figure 5G, red curve), locations where the CV lumen can be voluminous. These observations suggest that Ca^2+^ is not the limiting factor in CV and that growth cessation might be regulated by the availability of carbonate species.

The spatial relationship observed here between the vesicle membrane and the developing crystal surfaces provides complementary insights into a morphological control mechanism in coccolithophores (Figure 6). At initial stages of crystal growth, the CV membrane does not show regular areas of close proximity to the crystals, similar to previous observations in *P. carterae* and *E. huxleyi*.^17,18^ In these stages, the crystals are typically crystallographic with no curved surfaces, indicative of a relatively homogeneous growth environment (Figure 6A). However, at later stages (Figure 6B), we identified distinct proximity “bands” of intimate contact between the membrane and crystals, consistently positioned at the junctions between flat and curved surface-types. These points of high proximity appear to create functional boundaries within the CV (Figure 6C), effectively dividing it into subcompartments with different microenvironments, where growth occurs or halts. Thus, their presence at transition points between surface-types suggests a mechanism for localized control of the chemical environment, potentially regulating the availability of calcium and carbonate ions to specific regions of the growing crystal.

**Figure 6:**
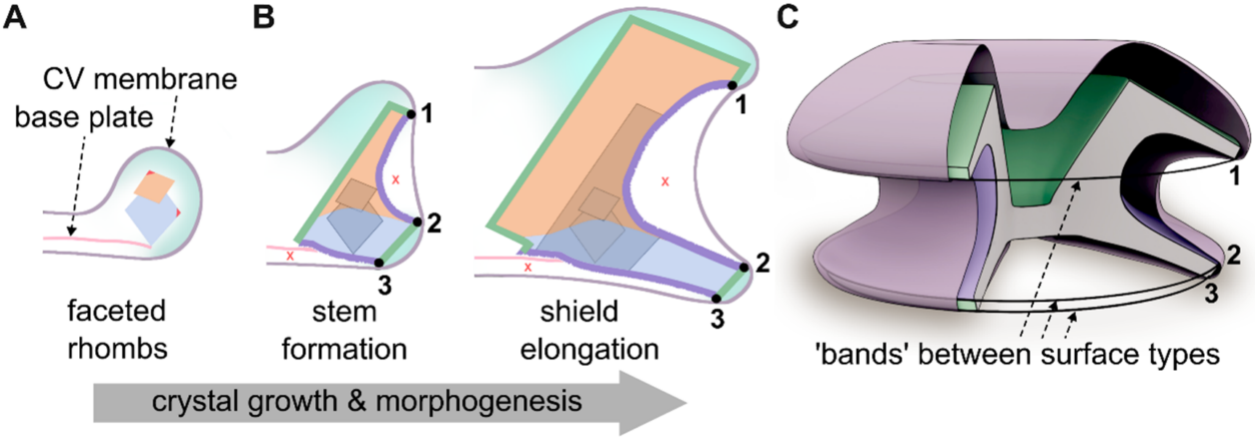
Model for membrane-mediated compartmentalization by structural “bands,” controlling growth regime. Suggested mechanism for membrane-mediated crystal morphogenesis, showing a schematic illustration of the primary growth motifs and crystal morphologies in *C. leptoporus* coccoliths, viewed along the BP plane. (A) Initial crystal formation occurs in a relatively isotropic environment, resulting in crystallographic rhombohedrons. Crystal units are depicted: V-unit (orange) and R-unit (blue), with red triangles indicating rough *c*-axis orientations. (B) High-proximity membrane-crystal interactions at specific areas in the coccolith are established (black circles), which form ‘bands’ around the structure (numbered 1 to 3). These bands break the relative symmetry of the CV lumen, creating distinct regions with divergent crystallization characteristics: (i) regions of growth (green gradients), promoting crystallographic and smooth surface development (green lines), and (ii) regions constraining crystal growth (red Xs), generating curved and textured surfaces (purple lines). The previous developmental stages are shown at each stage in gray, demonstrating the minimal growth at the purple surfaces relative to the green ones. (C) 3D cartoon of an entire CV with a mature coccolith inside. The proximity “bands” (1 to 3, corresponding to panel (B)) which lead to the compartmentalization of the lumen are colored in black. Different external crystal surfaces (flat or undulating) are colored according to panel (B).

This compartmentalization mechanism represents a significant advancement in our understanding of biomineralization control and differs from previously proposed models. Earlier studies on *E. huxleyi* and *P. carterae* suggested that crystal morphology might be controlled through persistent juxtaposition of the membrane, acting as a physical mold,^18^ or through spatially restricted ion diffusion leading to dendritic-like growth patterns.^17^ Our findings in *C. leptoporus* suggest a more nuanced mechanism, accommodating previous observations, where selective sealing at specific boundaries creates distinct subenvironments within a single vesicle, allowing for differential growth patterns without requiring membrane proximity to curved surfaces. The electron-dense complexes lining the luminal side of the CV membrane may play crucial roles in this regulation process, though they are not full ATPase complexes.^18^ While we could not definitively assign a specific function to these complexes due to insufficient resolution, they may represent a diverse array of proteins involved in multiple aspects of coccolith formation. Their ubiquitous nature suggests that they might translocate throughout the CV during different stages of coccolith development, potentially contributing to the temporal regulation of crystal growth by modulating the local chemical environment, as needed. In addition, the fact that cytoskeletal elements were not detected in contact with the CV, even though they were proposed to affect coccolith formation,^21,22^ points to an indirect role in coccolith morphogenesis.

Our results raise the question of the generality of this morphogenesis strategy, not only in coccolithophores but also in other organisms that produce crystals intracellularly within confined, membrane-bound organelles. Since high-resolution structural information is available for some of these cases, it is interesting to highlight those in which membrane interactions might play a role and cases where it is unlikely. In the production of holococcoliths, coccoliths produced in the haploid life-cycle of some coccolithophore cells and are composed of individual isotropic calcite rhombohedra that assemble into a larger structure,^31^ in situ works demonstrated that the individual crystals develop in a voluminous CV environment, where the crystals are further away from the membrane and no bounding “band” can be present, facilitating only the rhombohedral habit.^32,33^

Another important case is the magnetite crystals of magnetotactic bacteria that typically exhibit faceted morphology and, similar to early stage coccoliths, appear to develop in an environment that does not restrict their growth.^34,35^ Interestingly, some magnetotactic bacteria species produce “bullet”-shaped crystals with faceted tips and curved trailing surfaces.^36^ It will be interesting to investigate and see whether the membrane exhibits a high proximity to junction points between different surface-types during crystal morphogenesis. Other cases are guanine and hemozoin single crystals, whose growth occurs mostly within an ellipsoid lumen. They too present crystallographic habits that differ from their solution-grown equivalents; however, in these cases, it is the chemical composition of the solution (manifesting in the crystals themselves) or the guiding of templates (e.g., fibers) within the lumen that determines their shape and induces anisotropy.^37–40^ The membrane, however, plays no clear role in crystal morphogenesis, and indeed, in both cases, only crystallographic surfaces prevail during growth, and the crystals remain as simple polyhedra. The intricate silica cell-walls of diatoms, another group of unicellular phytoplankton, extend the role of membrane interactions in the morphogenesis of biological materials. Though the cell-walls are constructed from an amorphous material and are not crystalline, they have well-defined patterns and structural motifs. It was shown that at least some of their morphological features are driven by membranal molding,^41^ and the underlying chemical process might be a reaction-diffusion process facilitated by heterogeneous chemical conditions.^42^

## Conclusions

The findings presented here contribute to the understanding of morphogenesis control in coccoliths, where distinct membrane-crystal contact areas produce distinct subcompartments within a single vesicle. These contact areas appear to form contiguous proximity “bands” around the growing coccolith at junctions between crystallographic and noncrystallographic surfaces, establishing boundaries that may locally regulate crystal growth versus growth-inhibition. This compartmentalization strategy advances beyond previously proposed models, demonstrating how complex, reproducible crystal morphologies can be achieved without continuous membrane molding. Mechanistically, it alludes to a way by which biology acts to reduce the available degrees of freedom of the biomineralization process, potentially making it more robust against perturbations and thus less chaotic. Nevertheless, key questions remain open regarding the precise chemical composition within each subcompartment, the spatial distribution of ion transporters on the CV membrane, the mechanism responsible for membrane proximity at specific junction points, and the role of organic macromolecules in this compartmentalized growth process.

## Supplementary Material



## Data Availability

Relevant data is in the main text and Supporting Information. Additional data will be made available on request.

## Notes

Materials and Methods, SEM images of different surface-types in coccoliths, AFM surface scans of extracted coccoliths, cryo-FIB-SEM images, high-resolution cryo-TEM tomography images of internal coccoliths, FSCs for the 3D classes of subtomogram averaging, cryo-nano-XRF line profiles for different elements inside the CV (PDF)
