# Supplementary material for: Spatial Relations between Coccoliths and Their Confining Membrane During Crystal Morphogenesis
Source: J Am Chem Soc. 2026 Mar 24;148(13):14526–34. doi: 10.1021/jacs.6c02151 (PMC13067266; doi:10.1021/jacs.6c02151)
Supplement: Supplementary file 1 [file ja6c02151_si_001.pdf]

Supporting Information

Spatial Relations between Coccoliths and Their Confining Membrane During Crystal Morphogenesis

Emanuel M. Avrahami, Dmitry Karpov, Lior Aram, Nadav Elad, Razi Safadi, Irit Rosenhek-Goldian, Xiao-Meng Sui, Diede de Haan, Neta Varsano, Sidney R. Cohen, Igor Zlotnikov, Assaf Gal

This PDF file includes:

Materials and Methods

Figures S1 to S6

References

Materials and Methods

Cell cultures

Calcidiscus leptoporus (strain RCC1130) cultures were obtained from the Roscoff Culture Collection (www.roscoff-culture-collection.org) and grown in seawater (50 mL) from the Mediterranean Sea, adjusted to 3.5% salinity and supplemented with f/2 nutrient recipe.[1] Growth was maintained at 18 °C, with a lighting regime of 16 h light and 8 h dark. In these conditions, cultures show a typical growth curve over ~28 days, reaching maximal concentrations of 2-3x10^5 cells/mL.

Coccolith isolation for *ex situ* imaging

A 50 mL culture of exponentially growing cells (~80,000-140,000 cells/mL) was centrifuged for 10 minutes at 2,000 g (18°C). After removing the supernatant, the cells were gently resuspended in 500 µL hypotonic solution, containing 100 mM Na_2CO_3 and supplemented with 0.06 vol% NaClO in MilliQ water; freshly made prior to each experiment, through careful pipetting. The basic pH (pH≈11) of this solution prevents the coccoliths from dissolving and, together with the NaClO, helps break down organic cellular debris.[2] The 500 µL volume was transferred to a new 1.5 mL Eppendorf tube and left at room temperature for 10 minutes to allow coccoliths to settle, leaving the less dense organic debris in suspension. Subsequently, 450 µL of supernatant was gently removed, and an equal volume of fresh hypotonic solution was added. This washing step was repeated twice more, for a total of three washes. From this resulting solution samples for SEM and AFM samples were made (see 'Scanning electron microscopy imaging' and 'Atomic force microscopy imaging' sections).

Scanning electron microscopy imaging

To visualize the morphology and surface of coccoliths in a scanning electron microscope (SEM), 4 µL of isolated coccoliths (prepared according to the section 'Coccolith isolation for *ex situ* imaging') were blotted on a Whatman® Nuclepore™ Track-Etched Membrane,

with pore diameter of 100 nm (Merck, USA). Next, the coccoliths were coated with 2.5 nm of Iridium (Safematic, Switzerland), and imaged in Sigma 500 SEM or Ultra 55 FEG (Zeiss, Germany), using the InLens detector operating at 3 kV, and a typical working distance range of 2.5-3.5 mm.

Correlative atomic force microscopy/ scanning electron microscopy imaging

For AFM imaging, 5 μL of isolated coccoliths (prepared according to the section 'Coccolith isolation for *ex situ* imaging') were first blotted on a polycarbonate Whatman® Nuclepore™ Track-Etched Membrane, with pore diameter of 100 nm (Merck, USA). The membrane was then placed on an aluminum stub using a conductive adhesive tape. For correlative AFM/SEM imaging, an AFSEM nano (Quantum Design) AFM was mounted in the chamber of an environmental scanning electron microscope (ESEM, Quattro-S, Thermo Fisher Scientific Inc.). The samples were placed on the ESEM sample stage and scanning was performed by the AFM head. Self-sensing probes (PRS 70x30 μm self-sensing silicon cantilever with silicon tip, SCL-Sensortech, Langenlois, Austria) were operated in tapping mode. To accommodate the AFSEM head, the working distance of the ESEM was kept at 17 mm. ESEM images were taken with the SE mode of a GSED detector, using a 10 kV acceleration voltage and an aperture size of 3.0 to reduce the charging on the surface of the insulating sample, nitrogen gas was introduced into the chamber at a pressure of 200-240 Pa. The SEM was used to rapidly identify the region of interest for accurate positioning of the AFM probe. AFM imaging was performed with the e-beam turned off.

Cell freeze-fracture and cryo-SEM imaging

Exponentially growing *C. leptoporus* cells were pelleted at 2,000 g for 10 minutes (18 °C), and the cell pellet was re-suspended in 20 μL of seawater. 2 μL of the dense cell suspension was sandwiched between two metal discs (3-mm diameter, 0.1-mm cavities) and high-pressure frozen using HPM10 (Bal-Tec, CA, USA). The frozen samples were transferred under cryogenic conditions to a BAF 60 freeze-fracture device (Leica

Microsystems, Germany). Samples were observed at −120 °C in an Ultra 55 SEM (Zeiss, Germany) by using a secondary electron in-lens detector and a backscattered electron in-lens detector.

Cell preparation for cryo-fixation

Exponentially growing *C. leptoporus* cultures (~80,000-140,000 cells/mL) were decalcified (i.e., removal of external coccoliths) at 18 °C. For this, a 50 mL culture was treated with ethylenediaminetetraacetic acid (EDTA, pH=8.0) at a final concentration of 5 mM. EDTA, a Ca^{2+} chelator, dissolves calcite without penetrating cell membranes, thus removing external coccoliths while preserving those inside the cell, within the coccolith vesicle (CV). Removal of external coccoliths is necessary for subsequent focused ion beam (FIB) milling, due to the thickness and uneven surfaces of the crystals, which drastically hinder the ability to mill a flat surface. The decalcification process is typically completed (i.e., full dissolution of external coccoliths) within 2-5 minutes with gentle agitation. Cells were then centrifuged for 10 min at 2,000 g (18 °C), the supernatant removed and, last, gently resuspended in fresh 2 mL seawater supplemented with f/2. From this solution of concentrated decalcified cells, samples were immediately taken for subsequent cryo-fixation (detailed in ‘Cryo-fixation of cells for cryo-FIB milling).

Cryo-fixation of cells for cryo-FIB milling

From a decalcified culture (see ‘Cell preparation for cryo-fixation’), a volume of 4 μL was directly applied to glow-discharged Quantifoil® holey carbon R2/1 grids. Using the Leica GP EM, the grids were blotted for 3 sec from the backside (opposite the side of the sample) at 18 °C and 90% humidity, then immediately plunged into a liquid ethane bath. Finally, the grids were clipped into auto-grid rims (Thermo Fisher Scientific, USA) to allow their mounting into equipment necessary for further analysis.

FIB-SEM slice-and-view for large volume imaging

Cryo-fixed *C. leptoporus* cells on clipped grids were mounted into a SEM cryo holder under liquid nitrogen (LN) vapor in a vacuum cryo manipulation (VCM) loading station (Leica, Germany), and the holder was transferred to the LN-cooled Crossbeam 550 FIB-SEM microscope (Zeiss, Germany) using a vacuum cryo transfer unit (VCT) (Leica). Since we were interested in internal coccoliths (i.e., within the CV, within a cell), which we could not see prior to removing material from a cell and exposing them, we devised a simple approach for locating the coccolith. This approach included dividing the FIB milling into two parts: (i) rough milling until an internal coccolith was identified and (ii) more delicate milling for slice-and-view 3D volume imaging of the coccolith. The identification of an internal coccolith was made clear as its contrast (owing to its high Ca content) stood out from the background when observed via the energy selective backscattered (ESB) detector within the SEM (as seen in insets in panels C and D in Figure 2).

A protective layer of organometallic platinum precursor (Trimethyl(methylcyclopentadienyl)platinum(IV)) was deposited onto the grid for a total of 45 sec (30 sec on, 30 sec off, 15 sec on) using the gas injection system (GIS) built into the microscope, with the stage tilted at 35°. For FIB milling the stage was typically tilted to 25° and set to 5 mm working distance (to meet the coincidence point of the SEM and FIB beams). Rough milling was carried out with a FIB probe of 30 keV and 300 pA, until an internal coccolith was reached. Slice-and-view 3D imaging ('delicate milling') was carried out at 30 keV, 50 pA, and with 20 nm slice thickness, while SEM imaging was typically set to 1.5-2.0 keV, 40-50 pA, InLens/SE2 detector at 0.5-1 mixing (with majority of the signal coming from InLens), and 4-6 nm pixel size. SEM scanning was done at a scan speed of 1, with line-averaging noise reduction and N=60-80. All millings were set up in SmartFIB software (Zeiss). Brightness and contrast were manually adjusted for each sample. Data was collected until the edge of the CV was reached.

FIB-SEM data processing

Stripe artifacts (vertical milling stripes and horizontal charging stripes) were removed from the FIB-SEM data using combined wavelet-Fourier filtering in ImageJ. The image stack was aligned using Scale Invariant Feature Transform (SIFT) in ImageJ. 3D segmentations were performed in Amira 3D version 2021.2 (FEI, Thermo Fisher Scientific, USA). Coccolith structures were primarily segmented via contrast thresholding on the image stack collected from the ESB detector channel (due to the excellent contrast differences between the calcite and the organics), while CV membranes required manual segmentation.

Cryo-FIB lamellae milling for cryo-electron tomography

Cryo-fixed *C. leptoporus* cells on clipped grids were loaded into a Crossbeam 550 FIB/SEM dual beam microscope (Zeiss) using a VCM (Leica) equipped with a cryo stage. Before milling, grids were coated with a protective layer of organometallic trimethyl(methylcyclopentadienyl)platinum(IV)) by an *in situ* gas injection systems (GIS) (inside the chamber). GIS nozzle was opened for 30 sec, then closed for 15 sec, then opened in the same manner for two more additional times (total of 90 sec deposition time). The lamellae were milled at a 12° tilt relative to the grid plane, with a two-stage milling regime. First, rough milling (to ~1 μm thickness) involving two steps using the gallium ion beam at 30 kV and currents of 300 pA and 100 pA, and second, thinning to ~200 nm and polishing at a current of 50 pA.

Cryo-electron tomography

Cryo-electron tomography data was collected on cryo-lamellae from 12 cells. The tilt series were acquired using a Titan Krios G3i TEM (Thermo Fisher Scientific, Waltham, MA, USA), operating at 300 kV. Tilt series were recorded on a K3 direct detector (Gatan Inc., Pleasanton, CA, USA) installed behind a BioQuantum energy filter (Gatan Inc.,

Pleasanton, CA, USA), using a slit of 20 eV. All tilt series were recorded in counting mode at a nominal magnification of 33,000X or 53,000X, corresponding to a physical pixel size of 0.27 nm or 0.16 nm, respectively, using the dose-symmetric scheme starting from the lamella pre-tilt of −12° or 12° (dependent on grid orientation) and with 2° increments to acquire 51-53 tilts for each tomogram.[3] Tilt series were taken at a defocus range of 2–7 μm, with an objective aperture of 100 μm inserted. Tilt series were acquired using an automated low-dose procedure implemented in SerialEM v3.8[4] or with Thermo-Fisher Tomo5 software with a total dose of about 120 $e^-/Å^2$. Each tilt series was fractionated into 4 movie frames.

Subsequent tomogram alignment, using patch-tracking, and reconstruction were all done with IMOD.[5] Reconstructions were done using the back-projection algorithm or SIRT-like algorithm with ~70 iterations (empirically tested per dataset), when contrast was insufficient. Segmentations and three-dimensional visualizations of the reconstructed data were performed with the software Amira 3D version 2021.2 (Thermo Fisher Scientific, Waltham, MA, USA).

Subtomogram averaging for membrane-associated densities

Subtomogram averaging was performed using RELION 5.0-beta-3 software.[6] Movie frames (imaged at 2.7 Å/pixel) were motion-corrected, followed by CTF estimation within individual tilts using CTFFIND4[7] and tilt-series alignment using AreTomo[8]. Tomograms were reconstructed at 10 Å/pixel, and the membrane-associated complexes were manually picked from 4 tomograms, in which tilt series were well aligned and the complexes were clearly visible, using ArtiaX,[9] a plugin for UCSF ChimeraX.[10] 716 subtomograms were picked in total. The orientations associated with each coordinate were adjusted so that the Z axis pointed perpendicular to the membrane plane, and a random rotation around the new Z axis was applied (Euler angle manipulation was performed using a custom script). These adjustments to the Euler angles enabled the subsequent reconstruction of an initial reference map in RELION, while avoiding having the extracted subtomograms oriented similarly with respect to the missing wedge. Subtomograms were processed as 2D stacks in bin 3 (8.1 Å/pixel) and a 64^3 box size.

Reconstruction of an initial reference followed by 3D classification into 3 classes, with a mask of 250 Å diameter and 5° width prior applied on the tilt angle. Two classes that showed well-defined features were selected for subsequent 3D refinement using the same parameters. The two classes contained 499 (Fig. 4E, G right panel) and 157 (Fig. 4G left panel) subtomograms, and refined to 37 and 40 Å resolution, respectively (Fig. S6).

Sample handling and measurement conditions for synchrotron cryo-nano-XRF

Synchrotron measurements were carried out at the ID16A Nano-Imaging beamline at the European Synchrotron Radiation Facility (ESRF, Grenoble, France). The beamline is designed for cryogenic X-ray fluorescence nanoimaging and coherent hard X-ray phase contrast imaging. Beam focusing is achieved with two pairs of multilayer-coated Kirkpatrick-Baez (KB) mirrors and the beamline operates at discrete energies of 17.1 keV and 33.6 keV. The endstation can be operated under cryogenic conditions, which is advantageous for bioimaging because it helps preserve vitrified specimens and reduces radiation-induced damage. Cryogenic experiments are typically performed near 120 K.

Cryo-fixed specimens (cryo-fixation conditions were similar to those used for cryo-FIB/cryo-ET) on graphene-coated silicon nitride windows (5 x 5 mm window; 200 µm-thick silicon frame supporting a 1.5 × 1.5 mm, 500 nm-thick Si_3N_4 membrane) were handled in cryo-compatible boxes following workflows described in Karpov et al.[11] Silicon nitride membranes were then transferred to a cryo-manipulation workstation (EM VCM, Leica Microsystems, Germany), where they were kept submerged in LN2 throughout handling. Each membrane was mounted and clamped in a dedicated aluminum sample holder while still under LN2. The holder was subsequently introduced into the ID16A endstation through the docking port using a vacuum cryo-transfer system (EM VCT500, Leica Microsystems, Germany), which maintains the sample in a cryo-shielded environment under vacuum at approximately 110 K. After loading, the membrane was mounted in the high-vacuum chamber ($\approx 10^{-8}$ mbar) on a piezo-based nano-positioning stage equipped with six short-range actuators; positioning was monitored and regulated using a capacitive-sensor metrology system (twelve sensors). Throughout transfer and

measurement, the specimen temperature was maintained well below the devitrification threshold for biological samples.

Synchrotron X-ray fluorescence cryo-nanoimaging

For the cryo-nano-XRF tomography reported here, data were acquired at an excitation energy of 17.1 keV. The focused beam provided a nanoscale probe; in comparable beamline configurations, probe size characterization is performed using lithographically patterned test samples (e.g., thin-film metal features on silicon nitride membranes) and assessed by rapid orthogonal scans (knife-edge type analysis). An optical microscope integrated on the beamline was used to bring the specimen into the focal plane (depth-of-focus on the order of a few micrometers) and to position the region of interest in the X-ray beam.

Tomographic datasets were collected by raster scanning with a 35 nm step size and a dwell time of 20 ms per pixel. A total of 101 projections were acquired over 180° rotation, excluding the missing wedge angles in the range of ca. 40 degrees. XRF emission was recorded simultaneously on two detector systems positioned on opposite sides of the sample: a 16-element silicon drift detector array (ARDESIA-16)[12] and a 7-element silicon drift detector (Hitachi Vortex ME7). Under these conditions, the full tomographic acquisition required approximately 17 h.

Spectral processing, alignment, and 3D reconstruction

XRF spectra were processed using PyMCA, with per-pixel spectral fitting to extract elemental signals from each projection. Detector outputs were combined after initial preprocessing and used to generate 2D elemental maps for each angle. The resulting projection stack was then aligned to correct for drifts and angular-to-angular mis-registrations before tomographic reconstruction.

Three-dimensional elemental distributions were reconstructed from the aligned projections using a maximum-likelihood expectation-maximization (MLEM) algorithm,[13]

yielding volumetric maps of Ca (and other detected elements). Where quantitative reporting was required, elemental maps can be expressed as areal mass densities following a fundamental-parameter approach with instrument geometry calibrated against thin-film reference standard (AXO Dresden) and an assumed matrix composition/thickness (e.g., an ice matrix on the order of several micrometers), consistent with established ID16A cryo-nano-XRF processing workflow.[11]

3D visualizations of the XRF data were performed in Dragonfly 3D (version 2024) by Object Research Systems. The low intensity signal coming from the CV lumen was masked to create a new region of interest, with Look-Up Tables (LUTs) applied. This allowed to color the Ca signal based on pixel intensity. Opacity was adjusted for whole-volume presentation.

Line-profile analysis of intravesicular calcium gradients

To quantify spatial variations in Ca concentration within the coccolith vesicle lumen, we extracted line profiles oriented normal to the crystal surface from the reconstructed XRF tomography data. The analysis was performed on a representative axial slice through the reconstructed Ca volume, selected in an orientation that minimizes missing-wedge artifacts inherent to limited-angle tomographic acquisition.

Line-profile orientations were determined using an image-processing pipeline applied to a gradient-magnitude representation of the Ca distribution, as the gradient operation is an efficient way to identify edges. The gradient image was upsampled by a factor of four using bicubic interpolation and smoothed with sequential Gaussian filters (σ = 2.5 and 1.0 pixels) to reduce noise while preserving contour geometry that allowed more precise algorithmic extraction of the surface normals. The smoothed image was binarized using Otsu thresholding, and the crystal boundary was extracted by morphological skeletonization. An inside/outside mask was constructed by dilating the skeleton, filling enclosed regions, and eroding back to the original boundary thickness; this mask enabled consistent identification of the outward-facing direction at each measurement location.

Measurement positions along the crystal contour were selected manually. At each position, the local tangent direction was computed via principal component analysis of neighboring skeleton pixels within a 5-pixel radius. The surface normal was taken as the perpendicular to this tangent, with orientation adjusted to point outward based on the inside/outside mask. Line profiles were sampled along each normal, extending 4 pixels (140 nm) inward from the contour point and 10 pixels (350 nm) outward into the lumen, using original non-interpolated data. Multiple profiles were extracted to assess reproducibility of observed concentration gradients across different regions of the coccolith.

Supplementary Figures

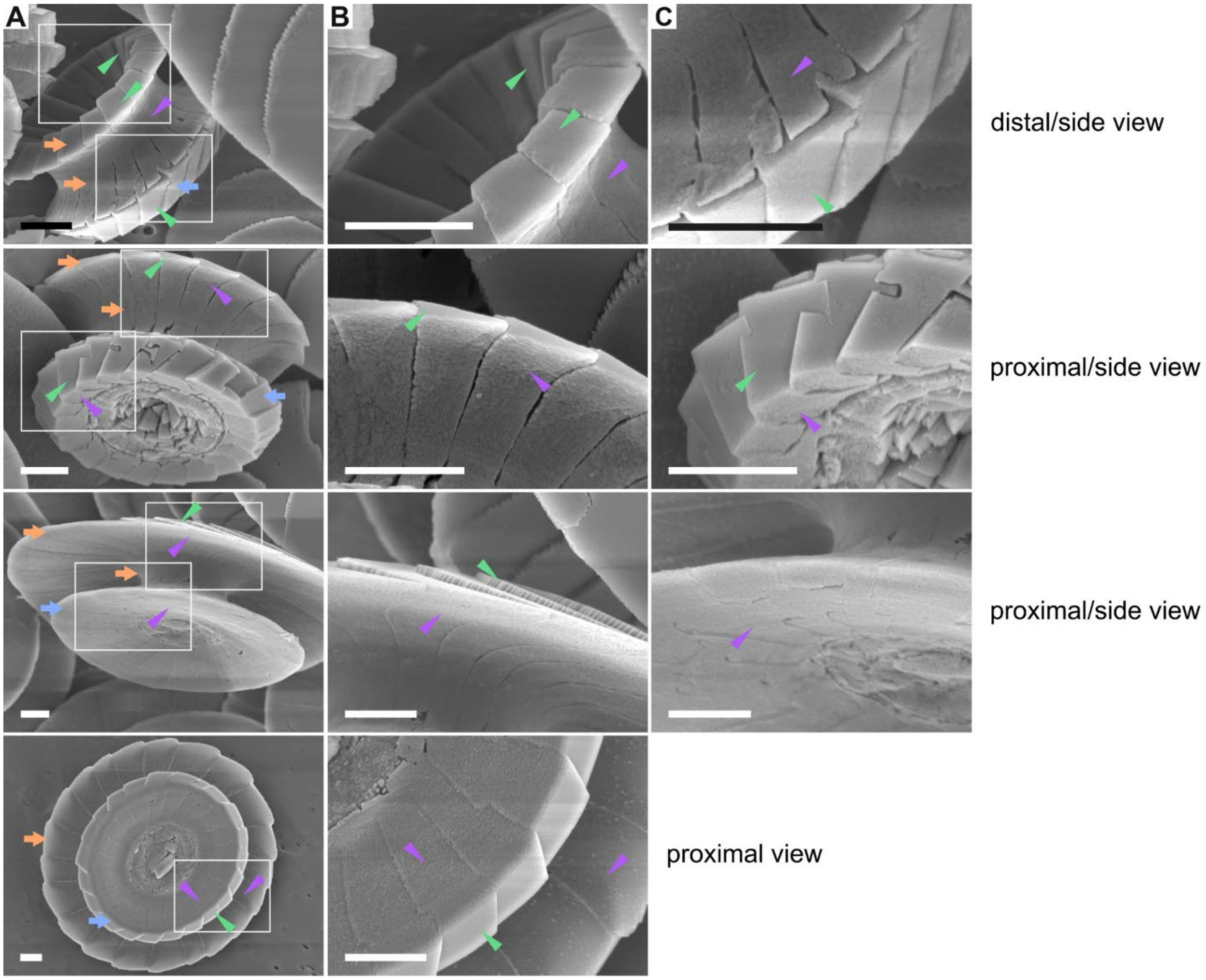

Fig. S1. SEM images of different surface-types in *C. leptoporus* coccoliths.

Column (**A**) shows four different coccoliths of various growth stages. Columns (**B**) and (**C**) show magnifications of the areas marked by white rectangles in (A). Orange and blue arrows indicate V-units and R-units, respectively. Green and purple arrowheads denote flat/crystallographic surfaces and curved surfaces, respectively. On the rightmost side is indicated the viewing direction of the coccolith. Scale bars, 500 nm.

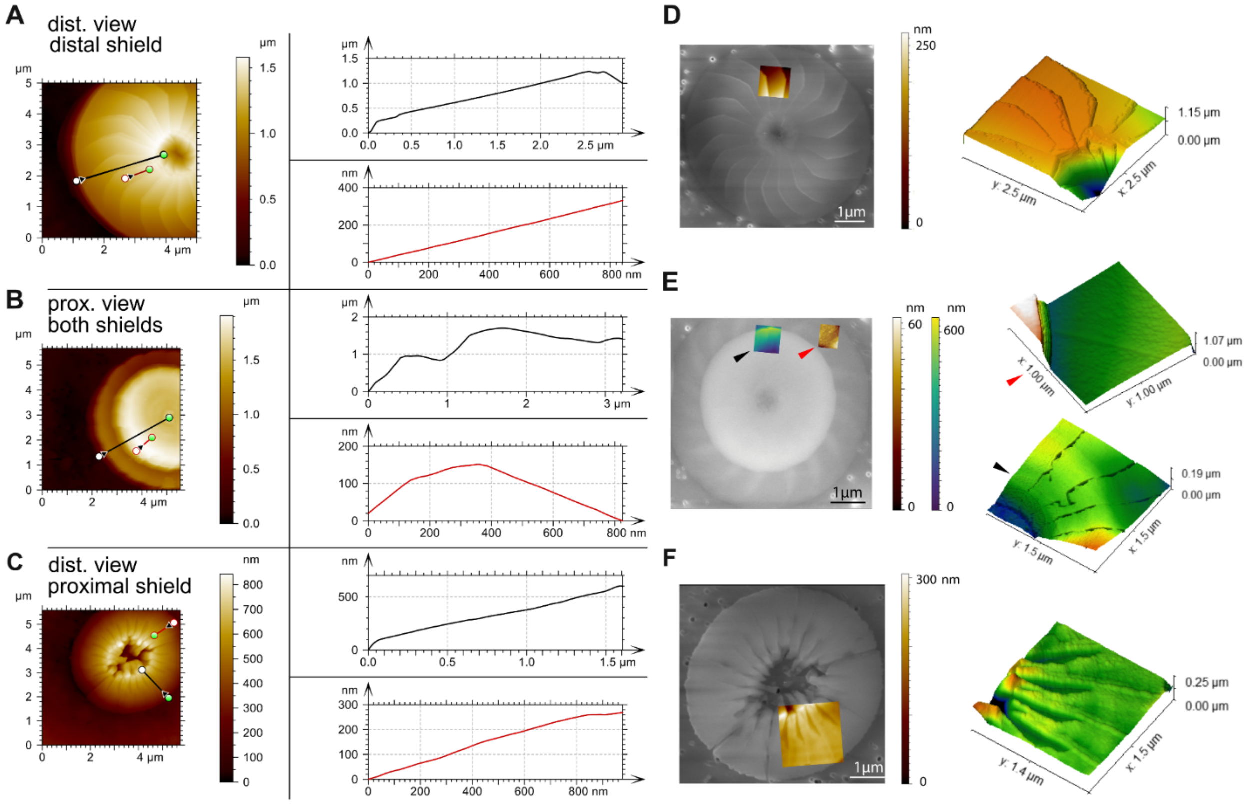

Fig. S2. AFM scans of extracted coccoliths – surface flatness/undulation and topography.

(**A**) to (**C**) Representative cross-section profiles, tracing from the outside of the element to the coccolith center, of the different surfaces of the two coccolith shields (viewing direction and shield identity of each surface is stated in each panel. Prox – proximal; dist – distal). Left, plan-view AFM image and height-scale color bars with long and short profile lines (black and red, respectively). Right, the corresponding color-matched lines. (**D**) to (**F**) Left, ESEM images of similar surface types and views as in (A) to (C), respectively, with overlayed AFM-scanned regions (colored rectangles on coccoliths). Note that in (E) there are two AFM images, indicated by black and red arrowheads on the ESEM images, with unique height scale bars. Right, corresponding 3D AFM visualizations of the different surface types, emphasizing the 3D topography of each region.

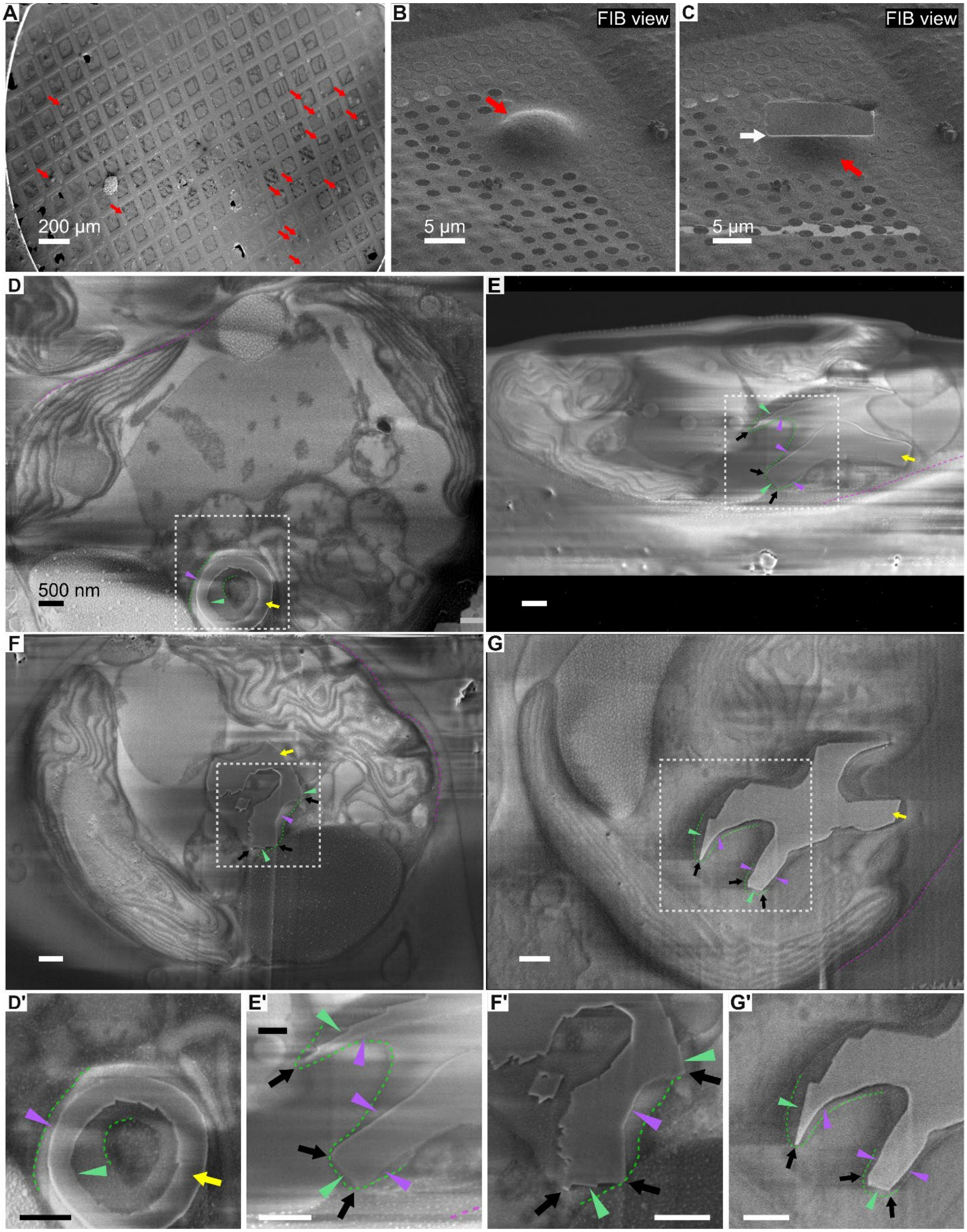

Fig. S3. Cryo-FIB-SEM of *C. leptoporus* cells containing internal coccoliths.

(**A**) Representative SEM image of a grid containing cryo-fixed cells (red arrows indicate several examples of cells), which have been decalcified. Image captured with InLens detector. (**B**) and (**C**) show a FIB view of a representative cryo-fixed cell before and after FIB milling, respectively. Red arrow indicated cell body and white arrow indicates the surface line of the milled cell. (**D**) to (**G**) Examples of four milled cells, showing the cell ultrastructure and internal coccolith (yellow arrows) within the coccolith vesicle. Several components, as discussed in the main text, are indicated schematically on the images: CV membrane, dashed green lines; cell membrane, dashed pink lines; crystallographic/flat surfaces, green arrowheads; curved surfaces, purple arrowheads; points of regular high proximity between CV membrane and the coccolith, black arrows. Dashed white squares indicate magnified areas shown in (D’) to (G’). Note that (D) shows a plan view of the coccolith ring, cutting through the stem region. Scale bars for (D) to (G) and (D’) to (G’), 500 nm.

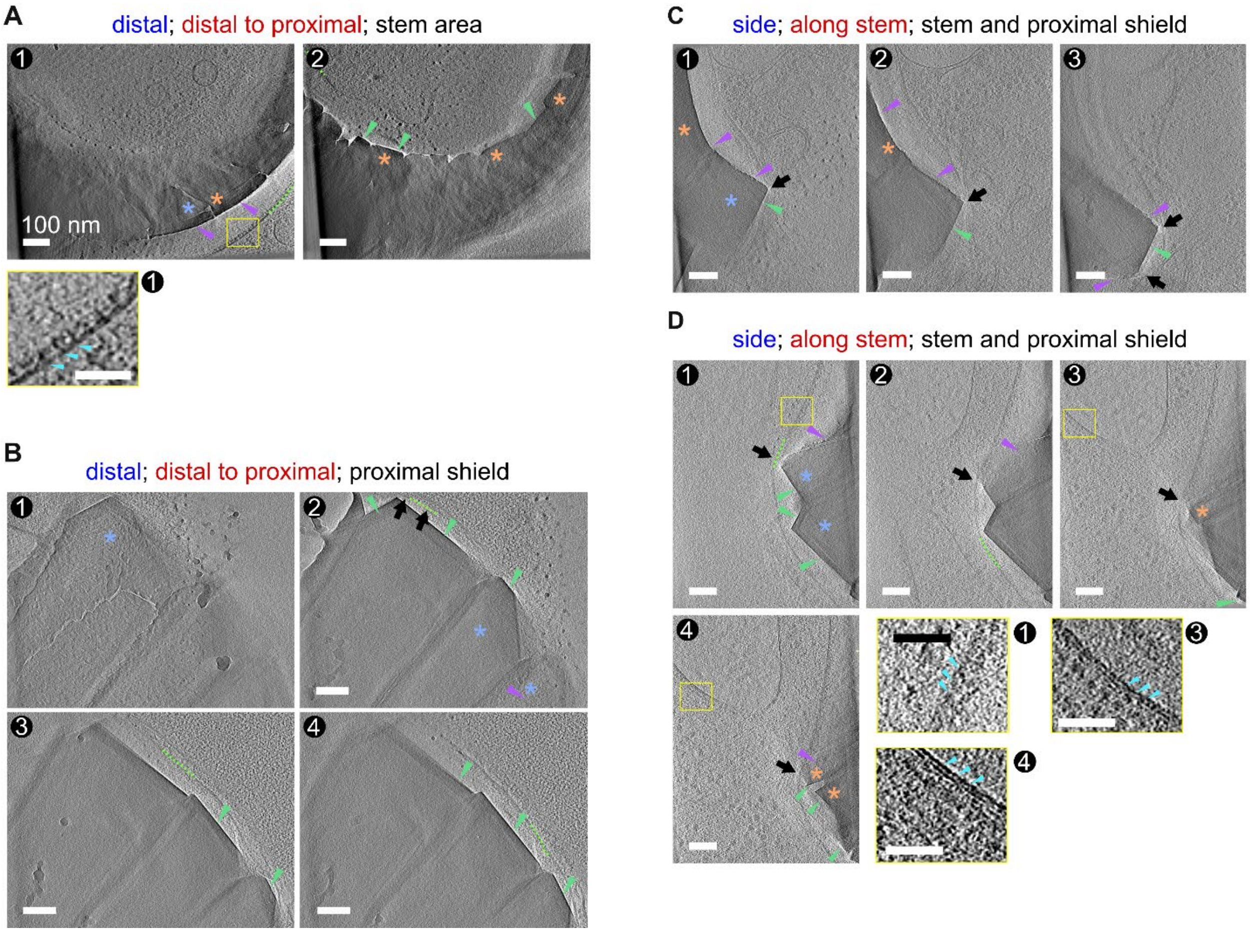

Fig. S4. High-resolution cryo-TEM tomography of internal coccoliths and their proximity to the CV membrane.

(**A**) to (**D**) Supplementary examples of datasets showing the relationship between the growing coccolith crystals, their surface type, and the CV membrane. Each panel shows a few key slices (numbers indicate order) along the datasets from the 200 nm-thick tomograms, with the important features indicated. At the top of each dataset is written its view direction (blue), the direction of movement along the dataset (red), and the observed coccolith element (black); all relative to the coccolith ring. Orange and blue asterisks indicate V-units and R-units, respectively. CV membrane is denoted by dashed green lines. Crystallographic/flat surfaces and curved surfaces are denoted by green and purple arrowheads, respectively. Black arrows indicate points of regular high proximity between the CV membrane and the coccolith crystals. Yellow rectangles on datasets mark electron-dense particles that are associated with the CV membrane. Scale bars, 100 nm. Number-matched magnifications of the rectangles (outlined in yellow) are shown next

to the relevant datasets. CV membrane is indicated by blue arrowheads. Magnified areas scale bars, 50 nm.

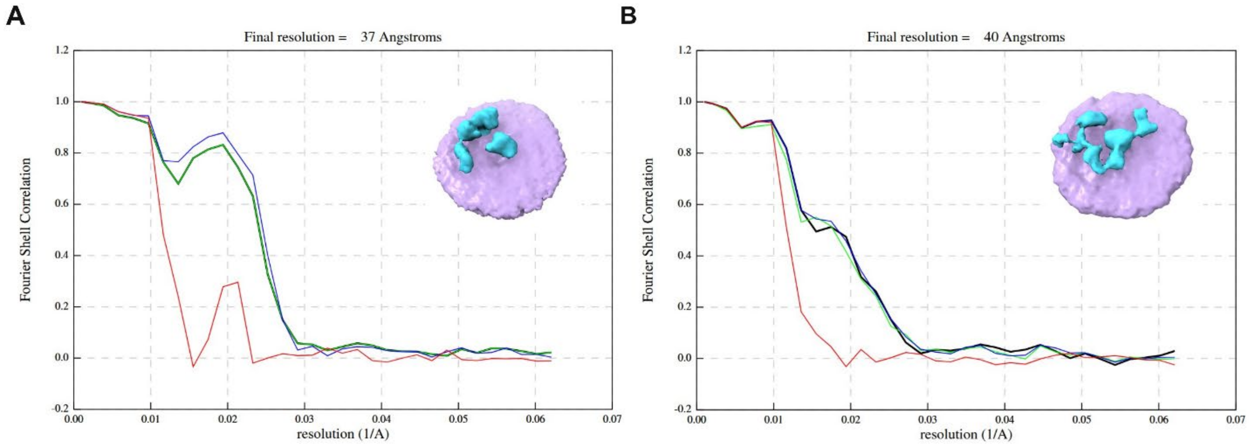

Fig. S5. Fourier shell correlations (FSCs) for the two 3D classes presented in Fig. 4.

(**A**) and (**B**) The two clusters identified. Curves include corrected FSC (black), unmasked maps FSC (green), masked maps FSC (blue) and phase randomized (red).

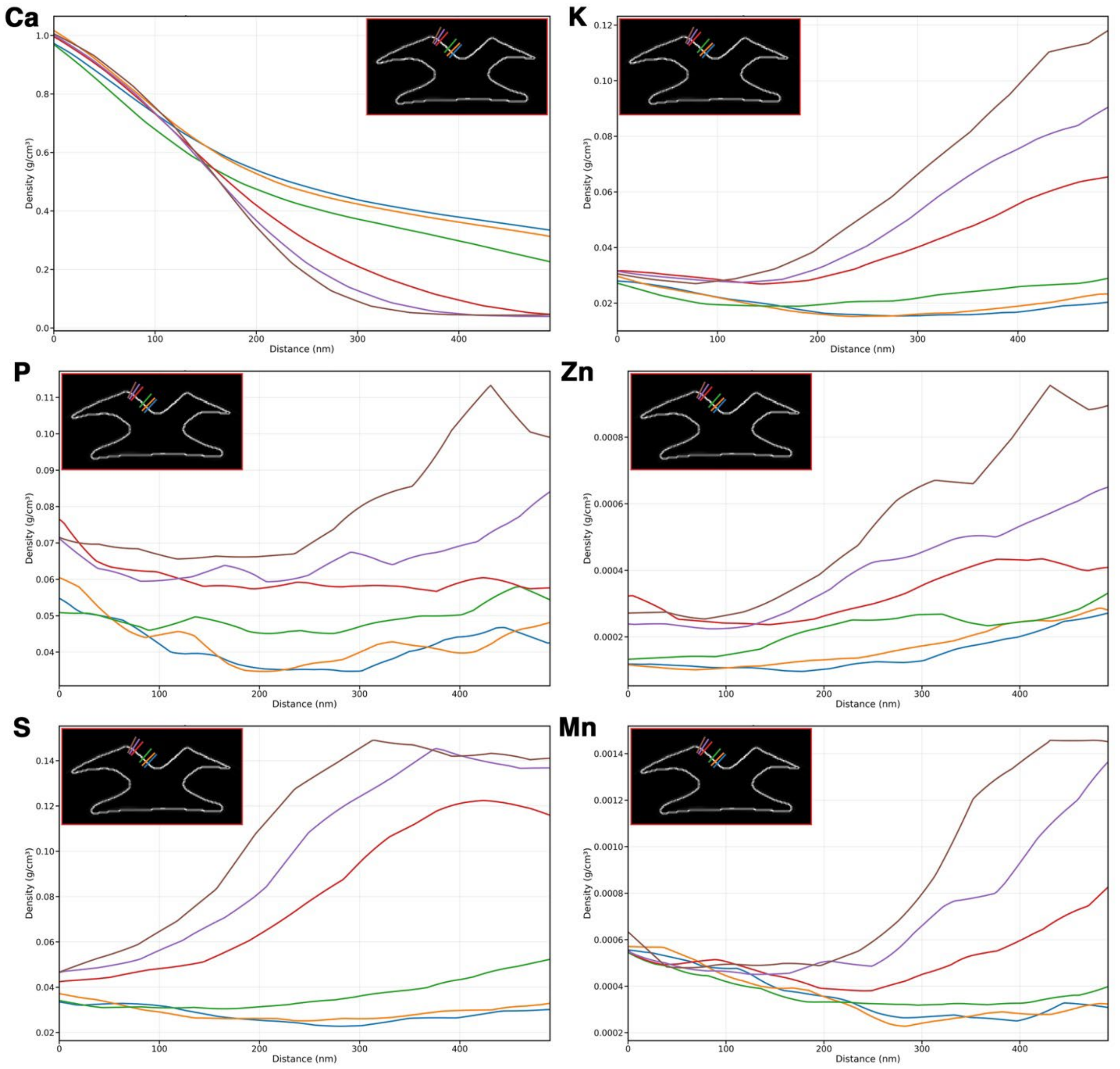

Fig. S6. Cryo-nano-XRF line profiles for different elements.

Examples of elemental line profiles measured across the coccolith boundary at multiple positions. Profiles were extracted along outward-pointing surface-normal lines (from inside the CV lumen toward the exterior) on the reconstructed slice, as described in 'Line-profile analysis of intravesicular calcium gradients', but for all elements detected. The edge of the coccolith surface is positioned 140 nm from the beginning of the line scan. Insets indicate the locations and orientations of the sampled line segments used to generate the profiles.

References

(1) Guillard, R. R. L. Culture of Phytoplankton for Feeding Marine Invertebrates. In *Culture of Marine Invertebrate Animals*; Springer, Boston, MA, 1975; pp 29–60.

(2) Avrahami, E. M.; Houben, L.; Aram, L.; Gal, A. Complex Morphologies of Biogenic Crystals Emerge from Anisotropic Growth of Symmetry-Related Facets. *Science.* **2022**, *376* (6590), 312–316.

(3) Hagen, W. J. H.; Wan, W.; Briggs, J. A. G. Implementation of a Cryo-Electron Tomography Tilt-Scheme Optimized for High Resolution Subtomogram Averaging. *J. Struct. Biol.* **2017**, *197* (2), 191–198.

(4) Mastronarde, D. N. Automated Electron Microscope Tomography Using Robust Prediction of Specimen Movements. *J. Struct. Biol.* **2005**, *152* (1), 36–51.

(5) Kremer, J. R.; Mastronarde, D. N.; McIntosh, J. R. R. Computer Visualization of Three-Dimensional Image Data Using IMOD. *J. Struct. Biol.* **1996**, *116* (1), 71–76.

(6) Burt, A.; Toader, B.; Warshamanage, R.; von Kügelgen, A.; Pyle, E.; Zivanov, J.; Kimanius, D.; Bharat, T. A. M.; Scheres, S. H. W. An Image Processing Pipeline for Electron Cryo-tomography in RELION5. *FEBS Open Bio* **2024**, *14* (11), 1788–1804.

(7) Rohou, A.; Grigorieff, N. CTFFIND4: Fast and Accurate Defocus Estimation from Electron Micrographs. *J. Struct. Biol.* **2015**, *192* (2), 216–221.

(8) Zheng, S.; Wolff, G.; Greenan, G.; Chen, Z.; Faas, F. G. A.; Bárcena, M.; Koster, A. J.; Cheng, Y.; Agard, D. A. AreTomo: An Integrated Software Package for Automated Marker-Free, Motion-Corrected Cryo-Electron Tomographic Alignment and Reconstruction. *J. Struct. Biol. X* **2022**, *6*, 100068.

(9) Ermel, U. H.; Arghittu, S. M.; Frangakis, A. S. ArtiaX: An Electron Tomography Toolbox for the Interactive Handling of sub-tomograms in UCSF ChimeraX. *Protein Science* **2022**, *31* (12), e4472.

(10) Goddard, T. D.; Huang, C. C.; Meng, E. C.; Pettersen, E. F.; Couch, G. S.; Morris, J. H.; Ferrin, T. E. UCSF ChimeraX: Meeting Modern Challenges in Visualization and Analysis. *Protein Science* **2018**, *27* (1), 14–25.

(11) Karpov, D.; Cuau, L.; Shishkov, R.; Gramaccioni, C.; Dallerba, E.; Schwehr, B. J.; Hackett, M. J.; Plush, S.; Massi, M.; Lerouge, F.; Cloetens, P.; Bohic, S. Cryo-Correlative Light and X-Ray Microscopies: Expanding the Intracellular Chemical Map. *bioRxiv*, 2025, 2025.05.23.655741.

(12) Utica, G.; Fabbrica, E.; Carminati, M.; Borghi, G.; Zorzi, N.; Ficorella, F.; Picciotto, A.; Allegretta, I.; Falkenberg, G.; Fiorini, C. ARDESIA-16: A 16-Channel SDD-Based Spectrometer for Energy Dispersive X-Ray Fluorescence Spectroscopy. *J. Instrum* **2021**, *16* (07), P07057.

(13) Dempster, A. P.; Laird, N. M.; Rubin, D. B. Maximum Likelihood from Incomplete Data Via the *EM* Algorithm. *J. R. Stat. Soc. Series B Stat. Methodol.* **1977**, *39* (1), 1–22.
